# Effect of plasmid replication deregulation via *inc* mutations on *E. coli* proteome & simple flux model analysis

**DOI:** 10.1186/s12934-015-0212-x

**Published:** 2015-03-08

**Authors:** Jonathan Meade, Patrick Bartlow, Ram Narayan Trivedi, Parvez Akhtar, Mohammad M Ataai, Saleem A Khan, Michael M Domach

**Affiliations:** Microbiology & Molecular Genetics, University of Pittsburgh School of Medicine, 15219 Pittsburgh, PA USA; Department Chemical Engineering, University of Pittsburgh, 15219 Pittsburgh, PA USA; Department Chemical Engineering, Carnegie Mellon University, 15213 Pittsburgh, PA USA

## Abstract

When the replication of a plasmid based on sucrose selection is deregulated via the *inc1* and *inc2* mutations, high copy numbers (7,000 or greater) are attained while the growth rate on minimal medium is negligibly affected. Adaptions were assumed to be required in order to sustain the growth rate. Proteomics indicated that indeed a number of adaptations occurred that included increased expression of ribosomal proteins and 2-oxoglutarate dehydrogenase. The operating space prescribed by a basic flux model that maintained phenotypic traits (e.g. growth, byproducts, etc.) within typical bounds of resolution was consistent with the flux implications of the proteomic changes.

## Background

The plasmid pNTC8485 replicates in *E. coli* and can be used as a platform for DNA vaccine production [1,2]. Antibiotic-free selection is used where a small antisense RNA is produced as opposed to a heterologous protein (e.g. β-lactamase) [3]. The small RNA binds to a chromosomally-encoded *sacB* transcript. Translation of the *sacB* transcript results in toxicity in a sucrose background unless the plasmid-encoded RNA is produced and then hybridizes with the transcript.

Previously, we reported on the effect of introducing the *inc1* and *inc2* mutations into this already high copy number plasmid [4]. The *inc1* and *inc2* mutations deregulate plasmid replication by further weakening the RNA I-RNA II interactions [5]. The reasons behind introducing these mutations were to: (i) increase the DNA yield achievable in processes that aim to produce DNA for vaccines or transfection purposes, (ii) assess the impact on growth, (iii) determine if the resulting amplification was similar to that observed for very low copy plasmids, and (iv) investigate whether integration or alterations in replication fidelity occurred. The second and third aspects were considered to shed new light on replication and the capacity of host cell metabolism in the context of producing very high copy number plasmids that do not produce heterologous protein.

After introducing the mutations, plasmid copy numbers (PCNs) ranging from 7,000 (early log phase) to 15,000 (onset of stationary phase) resulted when growth occurred on minimal medium. The growth phase and temperature influenced the PCN. Despite our starting point for copy number being orders of magnitude higher than Tomizawa and Som’s prior work [5], in LB medium the copy number was increased by a factor of 4- to 6-fold that was comparable to that achieved when the same mutations were introduced into very low copy number plasmids (ca. 3–30). Over a multi-generation batch cultivation cycle, no mutations were detected through sequencing the whole plasmid indicating that the host’s replication fidelity was maintained despite the high copy numbers. Also, the isolated plasmid was primarily super-coiled where topoisomers comprised the bulk of heterogeneity. Interestingly, the maximal specific growth rate of cells harboring the deregulated plasmid was not reduced when growth occurred in minimal medium [4].

The low impact on growth rate can be attributed, in part, to the differential expense of protein versus DNA synthesis. A DNA precursor has about three-times the mass of an amino acid. The ATP required per bond during DNA polymerization is about one-third of that needed for protein synthesis [6]. Thus, when combined, the ATP cost per unit mass is about one-tenth less for DNA polymerization than for protein polymerization. Additionally, as opposed to producing an antibiotic resistance marker protein (e.g. β-lactamase), which can comprise up to 18% of cellular protein [7], a short anti-sense RNA is expressed from the plasmid. On a per mass basis, RNA polymerization is also less ATP-intensive than the heterologous protein polymerization associated with conventional plasmids. Considering now the precursor burden, RNA breaks down faster in the cell than protein. Thus, the cycling RNA precursors can form their own pool that once established, less carbon is withdrawn from central metabolism due to rapid recycle. Thus, when compared at similar copy numbers, the precursor or carbon “burden” associated with the *inc* mutant plasmids can be envisioned to be lower than one would expect for conventional β-lactamase expressing plasmids.

Nonetheless, maintaining copies number ranging from 7,000 to 15,000 for a 3740 base pair plasmid represents the addition of several or more genome equivalents of DNA to the cell (i.e. 7 10^3^ × 3.7 10^3^/4.6 10^6^ = 5.6). Thus, while the energetics associated with synthesizing and maintaining the deregulated, sucrose-selected plasmids can be estimated to be less expensive than that for a conventional plasmid, the nil impact on nil growth could nonetheless be enabled by metabolic and other adaptations. For example, the *de novo* synthesis of deoxyribonucleoside triphosphates can require about 10 ATPs per molecule. The transcription from some of the more than 7000 extra genes that are available in a transformed cell will produce RNA. This RNA will degrade and the breakdown products will have to be converted back to ribonucleoside triphosphates prior to repolymerization. Thus, a number of impacts can be envisioned in the aggregate albeit per plasmid, they appear to be less than that normally associated with conventional plasmids.

To investigate if significant adaptations occurred, we pursued a two-pronged approach: (i) flux modeling and (ii) determining the changes within the proteome. The aim of the modeling was to determine what metabolic flux scenarios would allow for the plasmid-containing cells to grow at the measured rate (0.2. h^−1^) subject to measurements and the associated resolution of the by-products and yield on glucose. Such feasible flux scenarios could assist with interpreting the changes in the proteome. Additionally, alterations in nonmetabolic systems could emerge in the proteomic results that could further illuminate how cells may adapt to deregulated plasmid synthesis.

## Methods

### Network model & flux scenario generation

**Figure 1:**
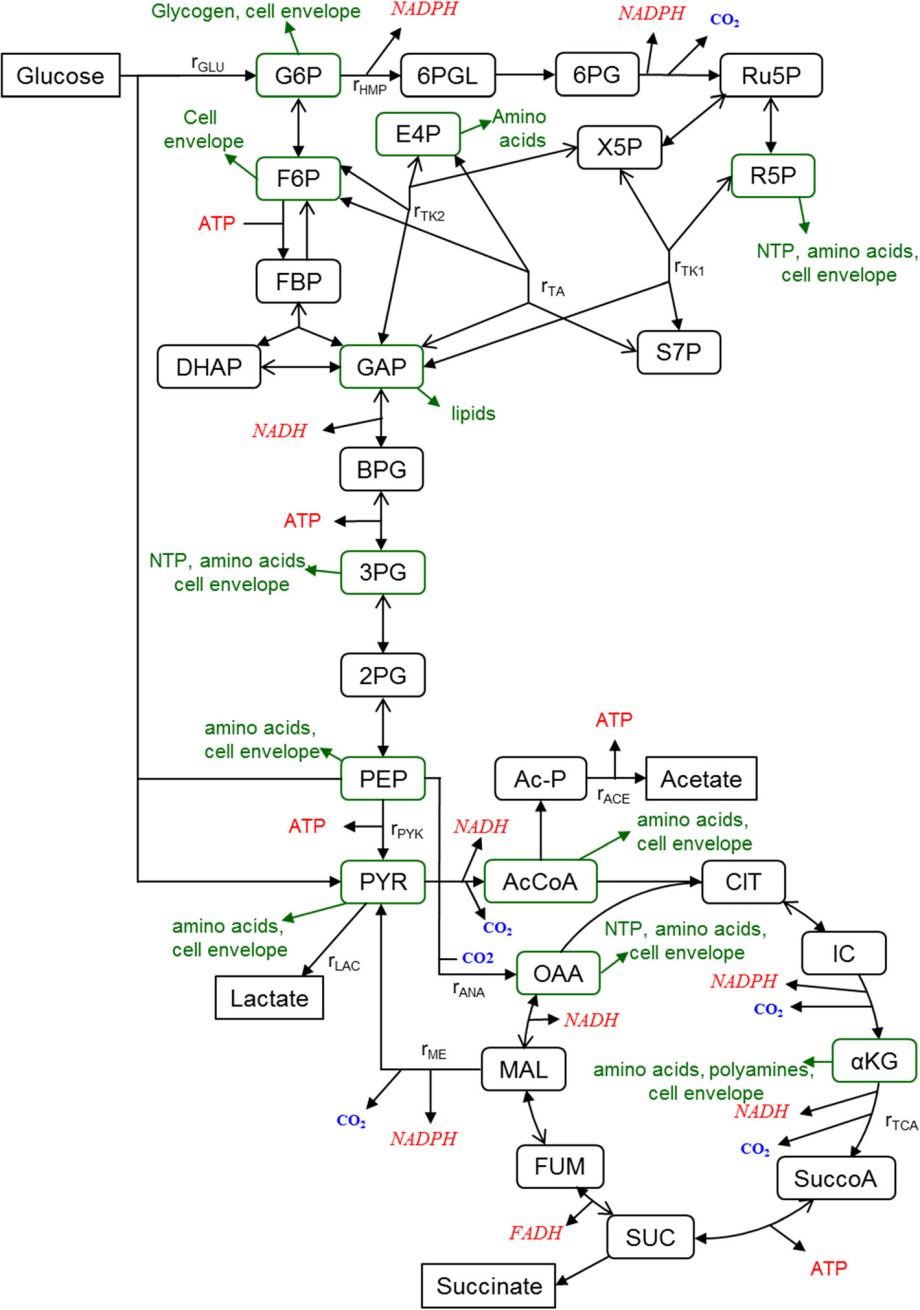
**Metabolic network for flux analysis.** Network used to model the central metabolism of *E. coli*. Metabolite abbreviations are αKG: α-ketoglutarate; 2PG: 2-phosphoglycerate; 3PG: 3-phosphoglycerate; 6PG: 6-phosphogluconate; 6PGL: 6-phosphogluconolactone; AcCoA: acetyl-coenzyme A; BPG: 1,3-bisphosphoglycerate; CIT: citrate; DHAP: dihydroxyacetone phosphate; E4P: erythrose 4-phosphate; F6P: fructose-6-phosphate; FBP: fructose-1,6-bisphosphate; FUM: fumarate; G6P: glucose-6-phosphate; GAP: glyceraldehyde phosphate; ICIT: isocitrate; MAL: malate; OAA: oxaloacetate; PEP: phosphoenolpyruvate; PYR: pyruvate; R5P: ribose-5-phosphate; Ru5P: ribulose-5-phosphate; S7P: sedoheptulose-7-phosphate; SUC: succinate; S-CoA: succinyl-coenzyme A; and X5P: xylulose-5-phosphate. Specific fluxes shown are those associated with glucose uptake (*r*_*GLU*_), hexose monophosphate pathway (r_*HMP*_), transaldolase (*r*_*TA*_), transketolase (*r*_*TK1*_ *, r*_*TK2*_), tricarboxylic acid cycle (*r*_*TCA*_), pyruvate kinase (*r*_*PYK*_), malic enzyme (*r*_*ME*_), anaplerotic replenishment of OAA (*r*_*ANA*_), and formation of lactate and acetate (*r*_*LAC*_ *, r*_*ACE*_).

The simple network model used is shown in Figure 1 and it is based on one previously used to assess the metabolic impact of plasmid production when β-lactamase is also synthesized [8]. The mainstream glycolytic and hexose monophosphate (HMP) pathways are represented as well as the tricarboxylic acid (TCA) cycle. Precursor diversions to biomacromolecules are also denoted where as previously done [8], the biosynthetic loads were based on published compositions and stoichiometries for precursor use [6].

Cells containing deregulated plasmid will experience increased DNA and RNA synthesis. The extra plasmid DNA will exert an increased precursor and ATP demand. However, the precursor load imposed by DNA is not normally high. For a cell growing at 0.2 h^−1^ with a cell mass yield (*Y*_*X/S*_) of 0.45 g cell/g glucose, the combined precursor load (i.e. total use of R5P, 3PG, and OAA) to deoxyribonucleotides can be estimated to be 0.04 mmol/g cell h, which is quite small compared to the glucose flux that is equal to 2.5 mmol/g h [6]. For 10 genome equivalents of DNA, the total precursor flux on a mmol/g cell h basis would be less than 10% of the glucose flux. The RNA l and RNA ll associated with plasmid replication as well as the small RNA that permits for selection via binding to the chromosomally-encoded *sacB* transcript will also be increasingly synthesized in plasmid-containing cells. However, rapid RNA turnover could allow for extensive recycling thereby reducing the net *de novo* synthesis burden on precursors. ATP use for repolymerization, however, could increase thereby lowering the ATP yield and increasing the apparent maintenance energy. As for wild-type cells, additional maintenance costs such as leaky membranes [9] might also prevail and possibly be exacerbated in transformed cells due to plasmid packing and partitioning effects [10].

One modeling approach would be to attempt to quantitatively account for all the aforementioned factors. For cells hosting β-lactamase encoding plasmids, such an approach provided yield and flux scenario predictions that spanned a range of promoter strengths and other uncertainties [8]. However, in this case, because there is no heterologous protein expression and thus plasmid production is relatively metabolically inexpensive from the carbon use standpoint, the problem of flux scenario generation can be first done in a simpler manner. Namely, the flux scenarios can be elucidated that can account for the similar phenotypes of the untransformed and transformed cells. This takes advantage of the fact that a number of constraints that define the phenotype are well known in terms of values and typical resolution. These constraints are *Y*_*X/S*_, byproduct fluxes, and growth rate.

These scenarios can be generated by initially assuming that precursor fluxes to DNA synthesis in both plasmid-free and -containing cells is not large compared to other fluxes. This still allows for an adaptive space by permitting total ATP production to vary subject to the fluxes must satisfy the phenotypic constraints and their range of resolution. That is, for all the flux scenarios, *Y*_*X/S*_ must fall into the experimentally resolved bounds prescribed by, for example, 0.42 ± 0.03 g cell/ g glucose. Essentially this amounts to finding flux scenarios that underlie macroscopically indistinguishable phenotypes (MIPs), which are defined by sets of largely similar measurements and their resolution bounds. These scenarios, in turn, prescribe the adaptive flux space that exists.

Some of the flux scenarios found may turn out to more likely correspond to plasmid-containing cells. For example, in some scenarios the value of the mass yield on ATP (*Y*_*X/ATP*_) might be lower. As noted before, more ATP production subject to fixed growth rate and other phenotypic features could be used to support the ATP costs associated with RNA repolymerization, DNA polymerization, and other processes that contribute to apparent maintenance. Thereafter, the impact of increasing the DNA biosynthetic load can then be tested and how the scenarios change can be determined.

Overall, the different flux scenarios associated with MIPs can be determined. The overarching idea is to see how far a “normal cell” can be stretched in terms of its fluxes while its phenotype in terms of yield, glucose uptake (i.e. *Y*_*X/S*_), and byproducts is fixed by measurements and resolution bounds. From a more mathematical perspective, a related idea is finding clonal solutions that differ by a small “epsilon” value from the true (or optimal) value [11]. Such solutions can be suboptimal, but they differ from the global optimal solution by a small, and often hard to distinguish margin.

Finding these flux scenarios entails solving

1a $$ MIN\ Z $$

*Subject to*

1b $$ Ar = b $$

1c $$ {r}_{lac} = 0 $$

*tight: r*_*ac*_ *= 1 or loose as*

1d $$ {r}_{ac}\ge 0.8 $$

1e $$ {r}_{ac}\le 1.2 $$

1f $$ {r}_{glu}\ge 2.469 $$

1g $$ {r}_{glu}\le 2.849 $$

1h $$ {r}_{ATP}\ge 20 $$

1i $$ {r}_{ATP}\le 28.6 $$

In the above, *Z* corresponds to an objective function such as minimizing a particular flux. The first constraint (1b) is the cell composition-based biosynthetic load and flux balance matrix that is typically used in this kind of problem and based on prior work [8]. Eqn (1c) accounts for the nil lactate formation that was measured in the case of plasmid-containing or -deficient cells [4]. The acetate production during exponential growth at a specific growth rate of 0.2 h^−1^ was found to be not significantly different in biological triplicates of untransformed and transformed cells and equal to about 1 mmol/g cell h with 20% variation [4]. As shown, either a tight formulation with acetate flux fixed at 1 mmol/g h or a looser formulation within resolution bounds can be used to see the effect on the feasible flux sets. The range of glucose flux allowed by Eqns (1f) and (1g) permits *Y*_*X/S*_ to be within the measured range of 0.42 ± 0.03 g cell/g glucose. Finally, the range permitted for ATP production allows for *Y*_*X/ATP*_ to fall in the range of 7 to 10 g cell/mol ATP for an assumed P/O ratio of 2.0 for NADH-linked oxidative phosphorylation and a value of 1.0 for FADH-linked processes.

If each flux is individually minimized (or maximized via MIN *– r*_*i*_), then the flux scenarios that populate the MIPs can be determined. While a mixed integer program could determine the extreme points [12], this problem is small enough such that iterating through each flux provides a means to classify the results in terms of which metabolic reactions are simultaneously maximized or minimized for each flux solution. The flux sets, in turn, can inform on how a cell could adapt its fluxes to maintain the specific growth rate by producing more or less ATP while also keeping by-products and other phenotypic features within experimentally resolvable ranges. Moreover, some of these flux scenarios may map to changes in the proteome where a subset of flux values and the level of associated enzymes in the proteome both change in a correlated manner. Those that do may then inform on how the host adapted to deregulated plasmid synthesis when the plasmid does not encode a heterologous protein.

### Proteomics

#### Host strains & plasmids

*E. coli* DH5α with *sacB* encoded in the chromosome (DH5α attλ::P_5/6 6/6_-RNA-IN- SacB, catR) and plasmid pNTC8485-EGFP (3,740 bp) were obtained from the Nature Technology Corporation (Lincoln, NE). The corresponding product identifiers are NTC-DV8485-LV and NTC-DVU-CC1. The *inc1* and *inc2* (single and double) mutations were introduced to pNTC8485-EGFP as previously described [4]. The *inc2* mutation was found to have a greater effect; hence, the proteomics experiments compared the untransformed host to that of a cell hosting the plasmid with an *inc2* mutation.

#### Quantitative proteomic analysis using iTraq

To generate samples for proteomic analysis, 500 ml cultures of host cells and those harboring a mutant (*inc2*) plasmid were grown in duplicate in M9 medium with 0.4 and 8% (by weight) glucose and sucrose. Two biological replicates of host and transformed cells were used. When the cultures reached mid-exponential phase (OD ~ 1.5), 250 ml of each culture was harvested by centrifugation (3300 rpm, 20 min, 4°C). Protein was extracted from culture pellets by the addition of 10 ml/mg pellet (wet weight) of buffer A (50 mM Tris–HCl, 50 mM NaCl, 0.5 mM EDTA, 5% glycerol, pH 7.5) plus 1 mg/ml lysozyme. Mixtures were incubated on ice for 10 minutes prior to sonication and centrifugation of debris. Supernatants were decanted, flash-frozen, and stored at −80°C.

The labeling of protein samples with iTRAQ tags, fractionation using two-dimensional liquid chromatography, identification of differentially expressed proteins by TOF/TOF tandem MS, and statistical analysis were performed as described in a prior report with only slight modification [13]. Briefly, 100 μg of total protein per sample was denatured by SDS, reduced using tris(2-carboxyethyl)phosphine, digested overnight with trypsin, and labeled with the 8-plex iTRAQ isobaric tags according to the iTRAQ manufacturer’s instructions (Applied Biosystems, Foster City, CA). Labeled protein samples (duplicates of host cell or *inc2* mutants harvested at mid-exponential or stationary phase) were pooled and half of the sample (400 μg) composed one 8-plex experimental run. Samples were fractionated by successive strong cation exchange and reverse phase chromatography prior to plate spotting and analysis using the ABI 4800Plus MALDI-TOF/TOF tandem MS system. Protein identification and relative abundance quantitation was performed using ProteinPilot software 3.0 (Applied Biosystems) and searches utilized the *E. coli* proteome database (Swiss-Prot/UniProt release 2011_11). The data was analyzed using replicate correlation analysis, principal component analysis, and a t-test. A change was considered to be significant if a protein in a cell containing a mutant plasmid was < 0.5 or > 2.0 that of the host, and *p* < 0.05. The experiments were conducted at the Genomics, Proteomics and Bioinformatics Center (Harvard University, Cambridge, MA) and we wish to thank Dr. Manoj Bhasin for his assistance with the data analysis. In this paper, only the exponential phase cells are compared because these are more amenable to flux analysis.

## Results

Table 1 summarizes the different flux sets associated with the MIP described by Eqn (1). All the sets shown correspond to constraining acetate production to be 1 mmol/g cell h. If the problem is instead solved using a wider byproduct range (*1d,e*), the results are similar as in Table 1 (not shown) in terms of scenario types, but the individual flux values range even further. Thus, Table 1 presents a more conservative or compressed flux space than that posed by 0.8 ≤ *r*_*ac*_ *≤ 1.*2 plus the other constraints (1f – 1i).

**Table 1:** Flux scenarios associated with macroscopically indistinguishable phenotypes*

**Objective**	***Y***_***x/s***_	***Y***_***x/ATP***_	***r***_***TCA***_	***r***_***PYK***_	***r***_***HMP***_	***r***_***ME***_	***r***_***TA***_	***r***_***TK1***_	***r***_***TK2***_	***r***_***ANA***_	***r***_***CO2***_
1. MIN *r*_*TCA*_	0.45	10	0.60	0.48	1.4	0	0.43	0.43	0.36	0.57	4.64
MIN *r*_*GLU*_											
MIN *r*_*ME*_											
MAX *r*_*HMP*_											
MAX *r*_*TA*_											
MAX *r*_*TK2*_											
MAX *r*_*4*_											
MAX *r*_*5*_											
2. MIN *r*_*PYK*_	0.44	10	0.77	0	0.99	0.63	0.29	0.29	0.22	1.2	4.12
**3. MAX** ***r***_***ME***_	**0.40**	**7.3**	**1.6**	**0**	**0.32**	**1.15**	**0.072**	**0.072**	**0**	**1.7**	**5.38**
**MAX** ***r***_***ANA***_											
**MIN** ***r***_***HMP***_											
**MIN** ***r***_***TA***_											
**MIN** ***r***_***TK2***_											
**MIN** ***r***_***4***_											
**MIN** ***r***_***5***_											
**4. MAX** ***r***_***TCA***_	**0.39**	**7.0**	**1.7**	**0.13**	**0.32**	**1.1**	**0.072**	**0.072**	**0**	**1.6**	**5.72**
**MAX** ***r***_***GLU***_											
5. MAX *r*_*PYK*_	0.39	7.0	1.4	0.93	1.0	0	0.30	0.30	0.23	0.57	6.59
MIN *r*_*ANA*_											

*Symbols: cell yield on glucose (g cell/g glucose), Y_x/s_; cell yield on ATP (g cell/mol ATP), Y_x/ATP_. See Figure 1 for *r*_*TCA*_ *, r*_*PYK*_ *, r*_*HMP*_ *, r*_*ME*_ *, r*_*TA*_ *, r*_*TK1*_ *, r*_*TK2*_, *and r*_*ANA*_; *r*_*CO2*_ is the net carbon dioxide flux. Flux units are mmol/g cell h. Bolded scenarios 3 and 4 indicate potential transformant solutions.

The flux plasticity is intriguingly high given that the resolution limitations of experimental measurements (growth rate, *Y*_*X/S*_*,* byproducts) are fairly narrow (~10% variation) and *Y*_*X/ATP*_ was constrained to be between 7 and 10 g cell/mol ATP. For example, the transaldolase (*r*_*TA*_) and transketolase (*r*_*TK1*_*,r*_*TK2*_) fluxes span ca 0.40 mmol/g h to that almost of a knockout (ca 0 mmol/ g h). The flux catalyzed by pyruvate kinase (*r*_*PYK*_) ranges from essentially knocked out (0) to 0.93 mmol/g cell h, which is about one third of the value of the glucose flux. Additionally, a range of solutions arise that offer overall ATP yields of 10, 7.3, or 7 g cell/mol ATP. Increasing the precursor loads for DNA by three-fold did not appreciably alter the number scenarios or the differences between the scenarios (not shown) as would be expected based on the prior remarks on the carbon demands of DNA synthesis.

**Figure 2:**
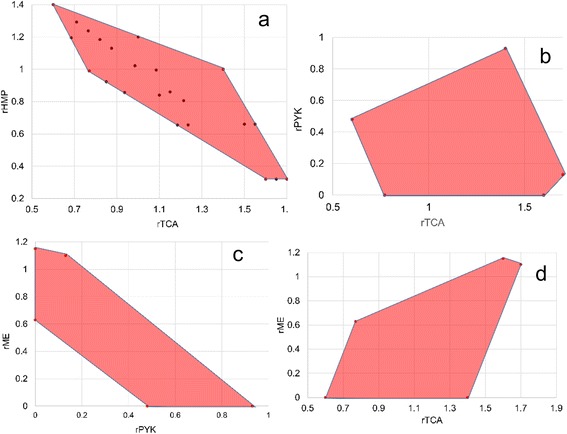
**Flux feasibility spaces from which the values of two fluxes can be chosen to resolve the two degrees of freedom.** The shaded region encloses feasible fluxes that support a growth rate of 0.2 h^-1^ and a glucose and ATP yield of 0.42 ± 0.3 g cell/g glucose and 8.5 g ± 1.5 g cell/mol ATP, respectively. In **(a)** different linear combinations of the solutions in Table 1 are shown to demonstrate that they fall on the edges or within the space defined by the extreme points listed individually in Table 1. Panels **(b)-(d)** show alternate flux pairs.

For the system, there are two degrees of freedom. Thus, two dimensional flux plots can further readily show the flux plasticity and well as the trends for two different fluxes. Such plots are shown in Figure 2. In Figure 2a different linear combination of the solutions in Table 1 are shown to lie on the edges or within the convex space. In Figure 2b, it is evident that a wide range of fluxes can occur through the TCA cycle and pyruvate kinase. An inverse relationship is seen between the fluxes through pyruvate kinase and malic enzyme in Figure 2c. In Figure 2d and the others, the shortest edge is bounded by scenarios 3 and 4.

Each scenario is now scrutinized further. Scenario 1 in the first row of Table 1 arises when the TCA cycle is minimized. This solution also corresponds to maximizing biomass production rate because the product of *Y*_*X/S*_ and the specific growth rate is maximized. Pyruvate kinase activity is mid-range and fluxes catalyzed by transketolase and transaldolase are at their highest values. This scenario is reminiscent of wild-type *E. coli* growing in a glucose background where TCA cycle repression occurs. Also, some workers use biomass production maximization as an objective function to predict wild-type fluxes [14,15], and this solution achieves that objective. Scenario 2 differs from Scenario 1 in that pyruvate kinase-catalyzed flux is zero and malic enzyme is active.

When compared to scenario 1, scenarios 2, 3 and 4 are similar in a number of respects. They all offer increased TCA cycle flux and reduced flux through pyruvate kinase. In comparison to scenario 1, scenarios 3 and 4 break out further by coordinately offering lower yields, increased TCA cycle flux, significantly reduced flux through pyruvate kinase, and substantially attenuated transaldolase- and transketolase-catalyzed fluxes. Thus, assuming that the maintenance energy of a transformant is higher and thus *Y*_*X/S*_ and *Y*_*X/ATP*_ are lower than the wild-type values, scenarios 3 and 4 could be candidates as flux solution components for the transformant.

Finally, the scenario 5 provides an alternative lower yield possibility. The pyruvate kinase-catalyzed flux is maximized while the TCA flux falls in between scenario 1 and scenarios 3 and 4. Overall, scenario 1 has higher yields, low TCA, and intermediate pyruvate kinase-catalyzed flux. In comparison two (3 and 4) have coordinated: (i) increased TCA flux, (ii) low pyruvate kinase-catalyzed flux, and (iii) diminished fluxes through transaldolase and transketolase. Two scenarios (2, 5) provide low (scenario 2) and high (scenario 5) pyruvate kinase flux alternatives to high and lower yields, respectively.

### Proteome contrast

**Figure 3:**
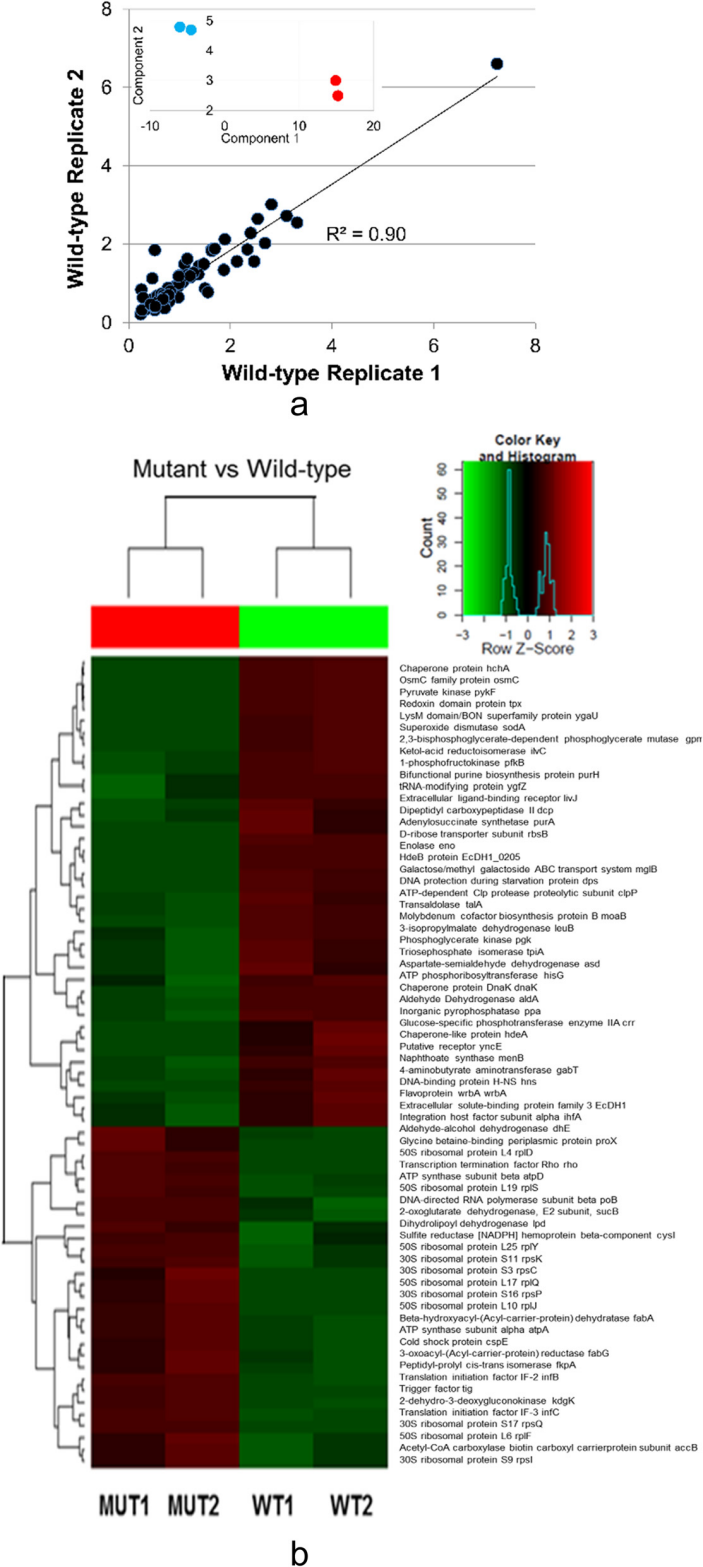
**Characteristics of the proteomics data. (a)** For the replicates sampled at mid-exponential growth phase, the protein abundances correlated well as shown for the untransformed cells. (Inset) Principal component analysis shows that the biological replicates group as well as separate from other conditions where red represents the transformed cells and aqua indicates the untransformed control. **(b)** Heat map view of the data for transformant replicates (left; MUT1 and MUT2) and untransformed (right; WT1 and WT2).

Sixty nine differentially expressed proteins were found based on screening at the level of *p* < 0.05 and a two-fold or more change. The correlation between the 69 proteins within the biological replicates was greater than 0.90 (see Figure 3a). When subjected to Principal Component Analysis, the biological replicates formed distinctive and separate clusters (see inset Figure 3a). Figure 3b provides a heat map representation as well as the distribution of Z-scores. Overall, the replicates provided reproducible results and pronounced differences were found between transformed and untransformed cells.

**Figure 4:**
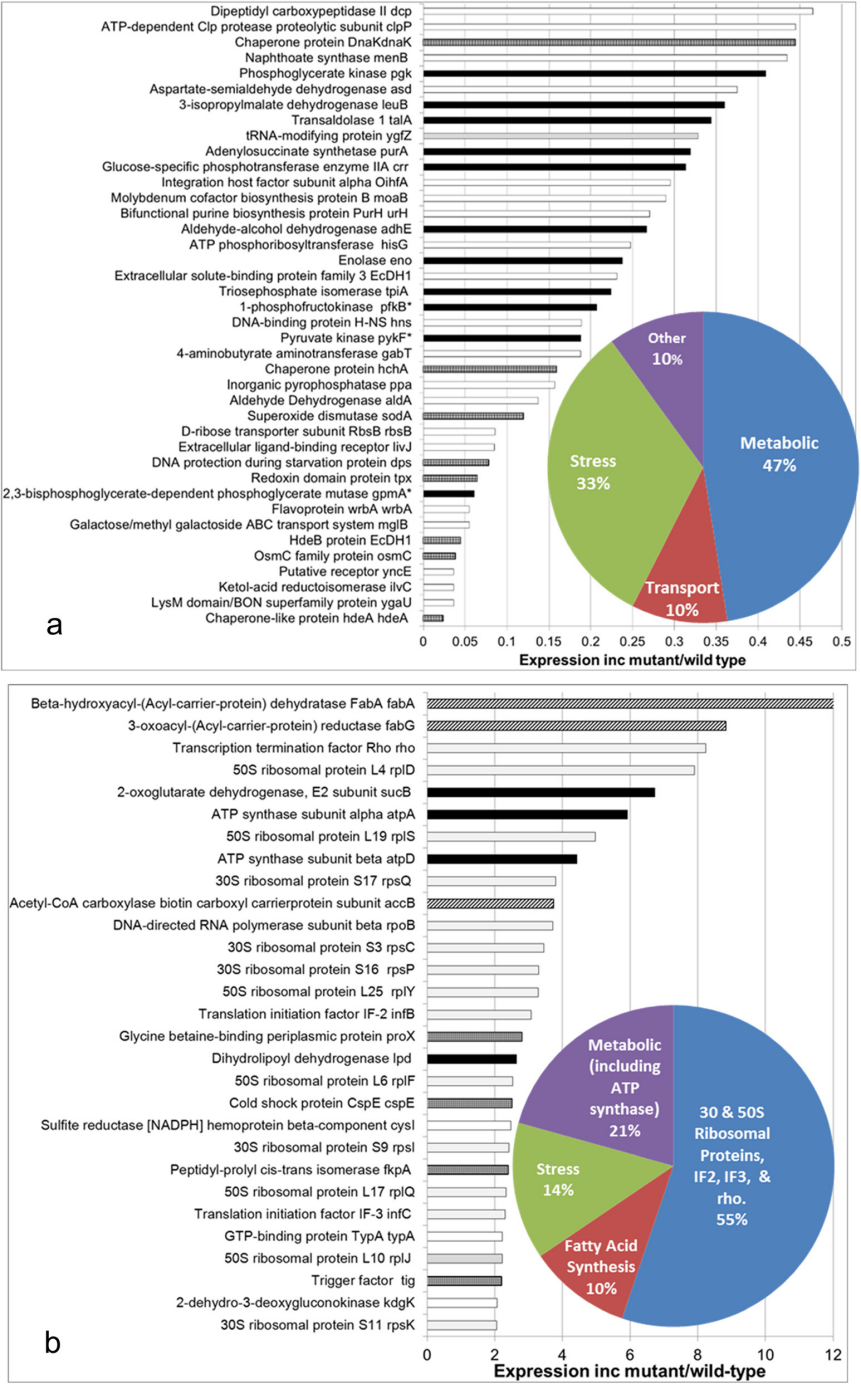
**Results of proteomic contrast of cells transformed with high copy mutant plasmids and nontransformed cells.** Downregulated **(a)** and up regulated **(b)** proteins are shown. In each category, a pie chart shown in inset breaks down the proteins into functional clusters. Functional grouping key: metabolic enzyme (solid black), translation-related (light gray), stress (medium gray/dots), and lipid-related (diagonal stripes). * denotes an isozyme or compensating enzyme activity exists.

The 69 differentially expressed proteins can be broken down into downregulated (Figure 4a) and upregulated (Figure 4b) groups. The downregulated and upregulated groups contain 40 and 29 proteins, respectively. Apart from the good correlation between biological replicates other confidence-adding features are present such as operon- and stochiometrically-linked proteins such as the *α* and *β* subunits of ATP synthase (3α:3β:1γ:1δ:1ε) [16] are found to be jointly upregulated. Functional groupings of proteins are also represented as pie chart insets in Figures 4a and b. One can see that ribosomal proteins comprising the 30 and 50S subunits as well as translational factors enrich the upregulated category. Another example is fatty acid/membrane synthesis enzymes are elevated such as the products of *fabG*, *fabA*, and *accB*. One would expect that if random errors accounted for most of the positives in the data, then the results would not be as extensively and tightly grouped by function.

Concerning metabolic enzymes, phosphofructokinase B is downregulated in the transformant (Figure 4a). To provide perspective, the activity of phosphofructokinase B is much less than that provided by phosphofructokinase A [17,18], which was found to be unchanged. Phosphoglycerate mutase is also considerably reduced in the transformant. To put this into context, *E. coli* has two phosphoglycerate mutase activities: 2,3-biphosphoglycerate-dependent (*gpmA*) and cofactor-independent (*gpmB*). In terms of protein abundance, both have been found to have comparable peak expression during growth in LB medium while the relative amounts of the two enzymes vary during batch growth [19]. The cofactor-independent enzyme was found to not be statistically different in the *inc2* mutant compared to the host. Pyruvate Kinase F, which has greater activity than the pyruvate kinase A [20], is downregulated in the transformant. The *talA* gene product Transaldolase 1 also emerged as downregulated.

In contrast to the above three enzymes, the E2 subunit of 2-oxoglutarate dehydrogenase is elevated in the transformant. Coinciding with this elevation is the increased expression of dihydyrolipoyl dehydrogenase, which constitutes the E3 component of the functional 2-oxoglutarate dehydrogenase complex [21]. Thus, the results show a correlated increase in E2 and E3. These and other proteins will be discussed further below.

## Discussion

**Figure 5:**
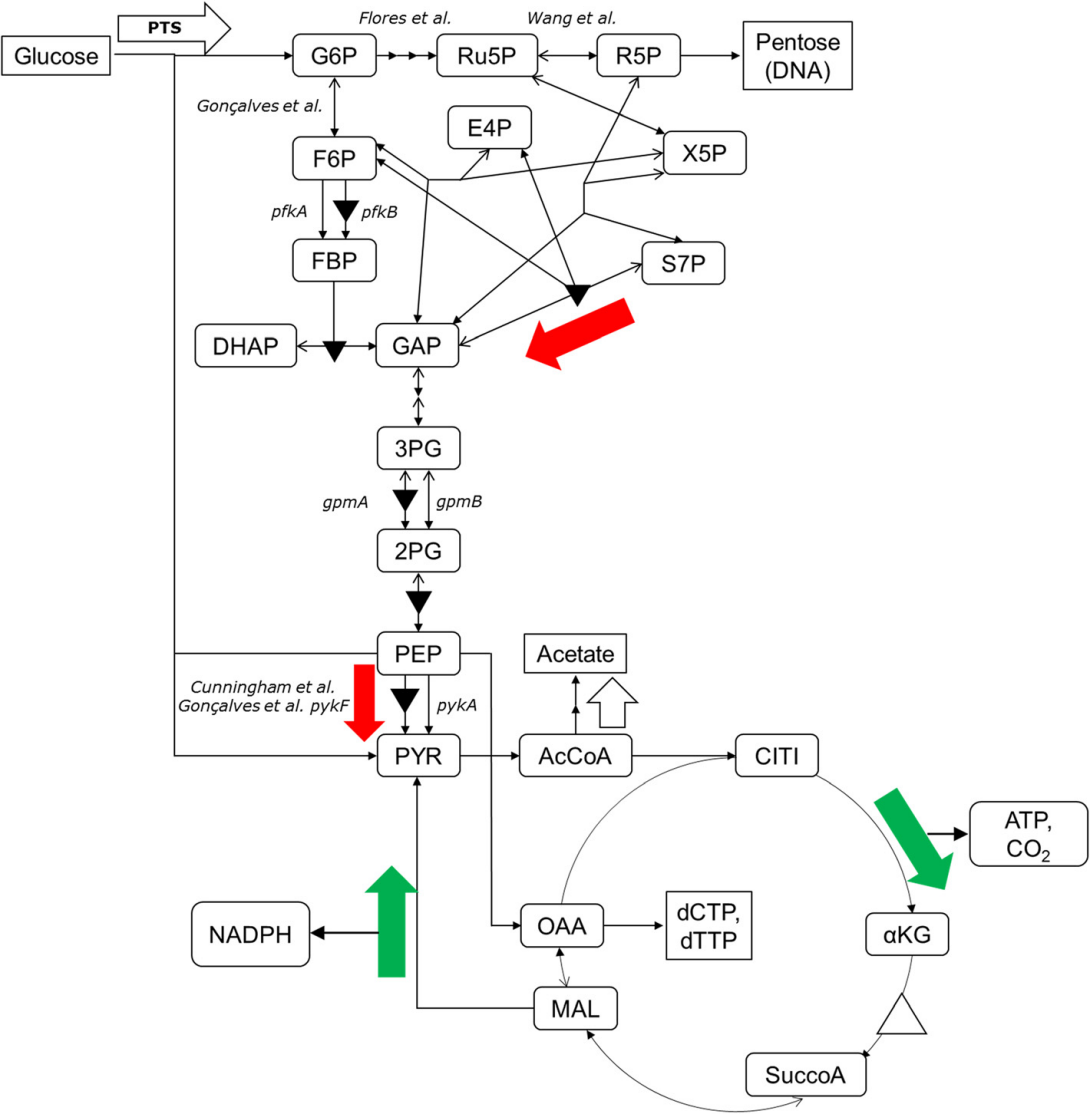
**Summary overlay of the current and prior studies for plasmid-containing cells.** The upregulated (apex-up, white triangles) and downregulated (apex-down, black triangles) metabolic enzymes found in this study as an adaptation to deregulated plasmid replication are shown. Also shown are annotations to enzymes that others have upregulated or knocked out in order to assess the effect on plasmid copy number or improve the tolerance to plasmid burden. Based on computed flux scenarios and projections from the proteomics, large arrows depict flux change possibilities for cells with deregulated plasmid replication as compared to untransformed cells; green, red, and white arrow colors correspond to increased, decreased, and not distinguishably different fluxes.

Figure 5 summarize the changes in metabolic enzymes we observed. The figure is also annotated with prior studies that manipulated individual enzymes in order to enhance PCN or to lower the burden imposed by plasmids [22-25]. Also, as will be developed below, some flux alteration projections and their metabolic consequences are also shown such as elevating ATP production. For example, Cunningham et al. knocked out pyruvate kinase in *E. coli* JM101 and found it to enhance the PCN [22]. Next to Cunningham et al. appears a triangle with a downward-directed apex indicative of the lowered expression of we found for pyruvate kinase F in the transformant (Figure 4a, *pykF* gene product). In this case, a reduction in flux as denoted by a red arrow is a clear cut consequence and also apart of some scenarios in Table 1.

While comparisons are not easily made due to the plasmid and other differences between studies, the prior reporting of upregulated TCA cycle enzymes corresponds, in part, to our findings. One study reported that transformants had increased levels of several TCA cycle enzymes (e.g. 2-oxoglutarate dehydrogenase) while pyruvate kinase was decreased [26]. We also find that the *talA* product Transaldolase 1is reduced in the transformant. Ow et al. [27] found that *talA* was one of the most highly downregulated genes in plasmid-containing *E. coli DH5α*. Likewise in chemostat studies with *E. coli* BL21, the comparison of specific enzyme activities indicated that transaldolase was more than two-fold reduced in a transformant [28]. Thus, a number of studies find that the same enzyme that interconverts glycolytic and hexose monophosphate pathway metabolites decreases in the presence of plasmids.

A large number of proteins related to ribosome assembly, processing, and functionality are upregulated in the transformant. These changes include elevated *rplD*, *rplF, rplJ, rplQ, rplS*, and *rplY* gene products. The L4, L6, L10, L17, L19, and L25 proteins are all associated with the 50S subunit where L4 participates in the early stages of ribosomal subunit assemblies [29]. The elevated gene products of *infB* and *infC*, IF2 and IF3, are factors involved in the early steps in initiating protein synthesis [30]. Within the category of ribosomal proteins, the products of *rpsC*, *rpsQ*, *rpsK*, *rpsI*, and *rpsP* are also increased in the transformant, which represents a cluster of proteins in the 30S subunit.

The clustered outcome for ribosomal factors may enable ribosomes to compete for shared metabolic and energetic resources or to maintain assembly and functionality in a situation of increased cytoplasmic crowding arising from deregulated plasmid replication. Finally, it has been estimated that approximately one-half of the transcription terminations in *E. coli* are Rho-dependent [31]. In the transformant, Rho is elevated suggesting that the control of read-through events may be increased.

Considering other protein classes, stress-related factors are either upregulated or downregulated in the transformant.

The gene products of *fkpA*, *tig, cspE,* and *proX* are represented in the upregulated category. The first two assist with protein folding where the *tig* gene product TF is thought to work in tandem as well as separate from DnaK and specialize in ribosomal and small (<20 kDa) proteins [32,33]. CspE and ProX, in turn, provide DNA/RNA melting and periplasmic binding capabilities for proline and betaine, respectively [34-36]. That some stress functions are elevated may also be reflected by increased levels of enzymes that fabricate fatty acids used for membrane construction (*fabA, fabB, accB*) [37-39]. Downregulated stress factors include the products of *dnaK*, *hns*, *hchA*, *sodA*, *dps*, *osmC*, *hdeA*, and *tpx*. As noted earlier, elevated TF may be able to provide needed protein chaperone activity. Some can be further grouped as (i) acid resistance factors (*hchA*, *hdeB*, and *hdeA*) [40,41] and (ii) oxidative stress factors (*sodA*, *tpx*) [42].

The link between the modeling (Table 1; Figure 2) and the proteomic outcomes can now be considered. The third and fourth scenarios in Table 1 that concurrently emphasize small transaldolase activity, high TCA flux, low flux through pyruvate kinase, and lower yields on glucose and ATP parallel the proteomic results assuming that a change in enzyme abundance translates to reduced flux. In some circumstances, prior work indicates that the assumption may be reasonable. For example, the E2 and E3 components of 2-oxoglutarate dehydrogenase are elevated in the transformant*.* 2-Oxoglutarate dehydrogenase is not normally highly expressed in *E. coli* [43,44] under glucose backgrounds and the reaction product (2-Oxoglutarate) is a feedback inhibitor of citrate synthase [45]. Thus, increasing 2-oxoglutarate dehydrogenase capacity can both reduce the feedback restriction at citrate synthase and increase the flux through 2-oxoglutarate dehydrogenase. That is, one can argue that this enzyme may have a significant flux control coefficient.

Considering that exerting flux control is plausible, then increased 2-oxoglutarate dehydrogenase capacity will manifest as increased flux through the TCA cycle. Such increased flux will result in greater ATP production. The outcome of greater glucose use and ATP production per cell mass points towards the lower cell mass yields on glucose and ATP associated with scenarios 3 and 4 in Table 1. More mechanistically, increased 2-oxoglutarate dehydrogenase capacity provides a means to cover the additional ATP use that will arise from extra RNA turnover and polymerization that was noted earlier to accompany the replication of the deregulated plasmid.

Such an additional ATP cost would also translate to a higher effective value of maintenance energy. This is interesting because Ow et al. determined that maximizing the maintenance energy as an objective provided the best fit to the fluxes determined for transformed *E. coli* [14]. Additionally, in our prior work on the effect of the *inc1* and *inc2* mutations, we found that an impact on growth rate occurred in a medium that was rich in precursors, but lacking in an energy source such as glucose [4]. This observation in conjunction with the modeling results (Table 1) further suggests that a cell transformed with the deregulated plasmid is tighter on energy (e.g. ATP) supply than anabolic carbon. Inducing greater 2-oxoglutarate dehydrogenase capacity may, in turn, help to supply the additional energy needed by providing great oxidative flux capacity and by reducing the feedback inhibition at the gateway step catalyzed by citrate synthase.

Looking further at the modeling results (Figure 2c) indicates that an inverse relationship exists between the fluxes catalyzed by pyruvate kinase and malic enzyme. Such an inverse relation was evident in the results of a ^13^C tracer and transcriptome study performed on a pyruvate kinase (*pykF*) knockout [46]. Additionally, some glycolytic enzymes were expressed at a lower level in the knockout. Thus, pyruvate kinase attenuation seems to be a common denominator in some *E. coli* strains for either adaption to the presence of plasmid [26] (Figure 4b) or for engineering enhanced plasmid production [22,24].

Another parallel emerges for the adaptations seen here and those found in other studies when pyruvate kinase was deliberately knocked out. During batch growth, carbon dioxide production was found to be greater in a knockout (PB25) than a wild-type JM101 strain by ~ 40% [47]. The flux scenarios in Table 1 that produce more ATP while having zero or very small pyruvate kinase flux (i.e. 3 and 4) also have the second and third highest values of CO_2_ flux. Overall, except for acetate production [47], the adaptations to the deregulated replication inferred from the proteomics or suggested by the adaptive flux modeling parallel what occurs in a pyruvate kinase knockout. The residual pyruvate kinase activity as opposed to the zero value of a full knockout may account for the acetate production of plasmid-containing cells. This acetate production can also lead to ATP formation at the substrate level adding to that produced by the upregulated TCA cycle.

Concerning transaldolase, this enzyme along with transketolases connects the NADPH-producing steps of the hexose monophosphate (HMP) pathway to glycolysis. The HMP pathway along with malic enzyme and isocitrate dehydrogenase supply the cell with NADPH along with some transhydrogenase-mediated conversion of NADH. The flux solutions in Table 1 indicate that scenarios exist such as 3 and 4 where malic enzyme can provide about one third of the total NADPH. Thus, in circumstances where malic enzyme is present and it can thermodynamically operate in the NADPH-producing direction, the hexose monophosphate pathway can be relieved somewhat from its task of NADPH supply. Note, however, apart from the absolute value of the flux into the HMP pathway (*r*_*HMP*_), the difference between this value and the return fluxes represents the net available for biosynthesis, which is also an important characteristic.

Other metabolic aspects now involve fluxes that may not have substantially changed. Phosphofructokinase A is comparable in the host and transformant whereas only the minor phosphofructokinase B activity was found to decrease. An irreversible feedback-regulated enzyme such as Phosphofructokinase A is generally not flux controlling [48]; hence, the total gateway glycolytic capacities of the mutant and host may not be substantially different. Also, it has been suggested that glycolysis has a lot of capacity and the flux is controlled through ATP turnover in an acceptor control-like manner [49]. Thus, the changes in glycolytic enzymes may have more to do with (i) altering the levels of metabolites and (ii) how fluxes may be distributed between glycolysis and the hexose monophosphate pathway than total fluxes of trioses. This is suggested further by most of the changes occur in enzymes that based on standard conditions, are viewed as catalyzing reversible reactions. Some maintain that reactions at or close to equilibrium provide a low degree of flux control [48]. Overall, one simple interpretation is extra carbon is sent to the TCA cycle in the transformant, which as mentioned earlier, can be processed by upregulated 2-oxoglutarate dehydrogenase. The high gain of ATP produced per differential mole of TCA flux can, in turn, contribute to the transformant’s increased ATP demand.

## Conclusions

In the context of antisense RNA selection for plasmid-containing cells and thus no plasmid-driven protein synthesis, *E. coli* growing on minimal media displays very similar phenotypic characteristics with respect to growth rate, yield on glucose, and byproducts when either transformed with a deregulated plasmid or not transformed. We conclude that within the narrow bounds of experimentally quantitated phenotypic descriptors, significant flux plasticity exists where apparently similar phenotypes can have quite different underlying fluxes. Moreover, we conclude that this plasticity appears to constitute an adaptive space that the cells used to contend with the altered energetics (e.g. RNA turnover/repolymerization) associated with deregulated plasmid replication. A subset of the solutions coordinately presented lower yield on glucose and ATP, low flux through pyruvate kinase, increased TCA cycle flux, and nil transaldolase flux. Proteomic experiments yielded results where increases or decreases in pyruvate kinase, transaldolase 1, and 2-oxoglutarate dehydrogenase paralleled the corresponding predicted fluxes in the subset. These changes and some relationships to prior studies are summarized in Figure 5.

Proteomic characterization also indicated that increases in 30 and 50S ribosomal proteins occurred in the transformant among other clustered changes. We suggest that the increased cytoplasmic crowding imposed by the thousands of plasmids may necessitate adaptations that improve the competiveness of binding and recognition associated with other large macromolecule-based processes. Apart from these particular results and conclusions, including a range for experimental measurements may also be useful for other flux balance analysis/optimization applications in order to fully map the flux solution space.
